# Optimizing surgical outcome of auricular keloid with a novel multimodal approach

**DOI:** 10.1038/s41598-022-07255-8

**Published:** 2022-03-03

**Authors:** Yi-Teng Hung, Shih-Min Lin, I-Shiang Tzeng, Chau Yee Ng

**Affiliations:** 1 Department of Dermatology, Chang Gung Memorial Hospital Linkou Main Branch, Taoyuan, Taiwan; 2 Department of Dermatologic Surgery, Chang Gung Memorial Hospital Linkou Main Branch, Taoyuan, Taiwan; 3 School of Medicine, College of Medicine, Chang Gung University, Taoyuan, Taiwan; 4 Department of Radiation Oncology, Chang Gung Memorial Hospital Linkou Main Branch, Taoyuan, Taiwan; 5 Department of Statistics, National Taipei University, Taipei, Taiwan

**Keywords:** Skin diseases, Skin manifestations

## Abstract

Various treatments are available for auricular keloids, but none has an absolute advantage. A practical and safe therapy to optimize the surgical outcome for auricular keloids is needed. We adopted a multimodal treatment of surgical enucleation, core fillet flap reconstruction, intraoperative corticosteroid injection, and immediate postoperative radiotherapy. There were no routine intralesional corticosteroid injections during follow-up. Keloid recurrences, complications, and risk factors for recurrences were analyzed. The outcome was compared with other published literatures. 45 auricular keloids were included in this study. 85.7% were female with an average age of 27.1 ± 7.5 years, and averaged size was 1.8 × 1.2 ± 0.9 × 0.6 cm. 71.1% were located at ear helix with 28.9% at the ear lobe. Nine keloids were classified as Chang-Park classification type I, 30 for type II, two for type III, and four for IV. The average radiation dosage was 1578.6 cGy. The recurrence rate was 6.7% at an average 24.1-month follow-up. There were no complications of surgery, radiotherapy, and intralesional corticosteroid injection. Our recurrence rate was lower than those in mono-adjuvant therapies of intraoperative corticosteroid injection or radiotherapy. This one-session multimodal approach optimizes treating auricular keloids with a low recurrence rate and minimal post-radiation and long-term corticosteroid injection-related complications.

## Introduction

Keloid is a wound healing complication with a continually growing scar following minor trauma, infection, burn, or inflammation^1^, and is characterized by aberrant growth of scar tissue extending beyond the previous wound margins. The ear is a common location of keloid formation, usually caused by ear piercing. Patients are often teenagers and young adults, whereby disfigured auricular keloids impose psychological stress and anxiety. Surgical excision is indicated for large, disfigured auricular keloids. However, surgical excision alone has a high recurrence rate up to 100%, suggesting the importance of adjuvant therapy^2^. The postoperative adjuvant therapies consist of intralesional corticosteroid injection, radiation, pressure therapy, cryotherapy therapy, topical mitomycin C, topical imiquimod, and intralesional 5-fluorouracil^3^. Nevertheless, there has been no standard regimen for auricular keloids to achieve the goal of a low recurrence rate, minimal side effects, and significant aesthetic improvement of auricular contour. Many of these treatments require multiple postoperative session visits. Therefore, the establishment of optimal multimodal therapy for auricular keloids is critical. In this study, we presented our experience in treating auricular keloids with a multimodal approach in a single session composed of surgical enucleation, core fillet flap reconstruction, intraoperative intralesional corticosteroid injection, and immediate postoperative radiotherapy.

## Methods

### Patient collection

We retrospectively reviewed the electronic medical database and photographs of all patients receiving auricle keloid surgery from a tertiary medical center from December 2017 to May 2021. Patients who received a multimodal therapy composed of surgical enucleation, core fillet flap reconstruction, intraoperative intralesional injection, and immediate postoperative radiotherapy were included for analyses. Patients with incomplete medical records or photography were excluded from this study.

A total of 45 auricular keloids from 35 patients were included. The clinical characteristics, including age, sex, previous history of keloid treatment, size and location of the auricular keloid, postoperative wound healing, recurrence, and follow-up periods, were recorded. We classified the auricle keloids with the modified Chang-Park classification based on clinical photographs^4^. Modified Chang-Park classification of auricular keloids consists of type I (pedunculated type), type II (sessile type with a single nodular pattern), type III (sessile type with a multi-nodular pattern), type IV (buried type), and type V (mixed type)^4^.

### Multimodal treatment protocol

#### Surgical method

The surgical method of enucleation and reconstruction with a core fillet flap was performed under local anesthesia (1% lidocaine with 1:100,000 epinephrine). The overlying skin of the keloid was dissected from the underlying keloid mass as a core fillet flap. Subsequently, the keloid core mass was enucleated, and bleeding was meticulously controlled by electrocauterization. The fillet flap is an axial pedicle flap, first introduced in reconstructing traumatic amputation^5^, utilizing the "spare parts" from the adjacent tissue. We used this method to reconstruct the wound after keloidectomy^6^. The excessive part was trimmed meticulously, and the surgical margin was approximated with 6–0 nylon sutures.

#### Intraoperative intralesional corticosteroid injection

Immediately after excision, triamcinolone acetonide (40 mg/mL) at a pure concentration was injected into the surrounding dermal skin with the volume given proportional to incision length (range, 0.1–0.5 mL). There were no postoperative injections during follow-up visits unless the patient has a sign of recurrence.

#### Radiotherapy

Keloids were irradiated with a 6-MeV electron beam of 15–18 Gy in three fractions by the linear accelerator (Varian Clinac® iX) performed at a 110-cm source-to-skin distance. The first fraction was completed within postoperative 24 hours, and the remaining two fractions were performed in the consecutive four days. The scar regions after excision were positioned and confirmed by the radiation oncologist. A 0.5-cm silicone bolus was used to improve the surface dose. The radiation field included the entire postoperative scar with a margin of 1 cm. The field was shaped by a unique cone and 0.8-cm customized cerebrum block.

### Therapeutic effect evaluation

Recurrence was defined as the presence of hypertrophic scar raised above the level of the adjacent skin shown in the clinical photographs or medical records. We also extracted the records of the wound healing time, total follow-up period, and postoperative complications. We further analyzed the clinical characteristics of patients, type of auricular keloids, and radiation dosage to determine the risk factors for keloid recurrences.

### Statistical analysis

Statistical analyses were performed with R software. The effect size calculated from a power analysis (Cohen’s *d* = 0.9) showed 42. Continuous variables were described with means and standard deviations, while categorical variables were presented as numbers and percentages. The variables between recurrence and non-recurrence groups were analyzed with Student's T-test, Chi-square test, and Mann–Whitney U test. A *p*-value < 0.05 was considered statistically significant.

### Ethics and groups

This study was approved by the Chang Gung Medical Foundation Institutional Review Board (IRB no. 202100809B0). All methods were carried out in accordance with relevant guidelines and regulations. Informed consent was waived by Chang Gung Medical Foundation IRB.

## Results

### Clinical characteristics

**Figure 1:**
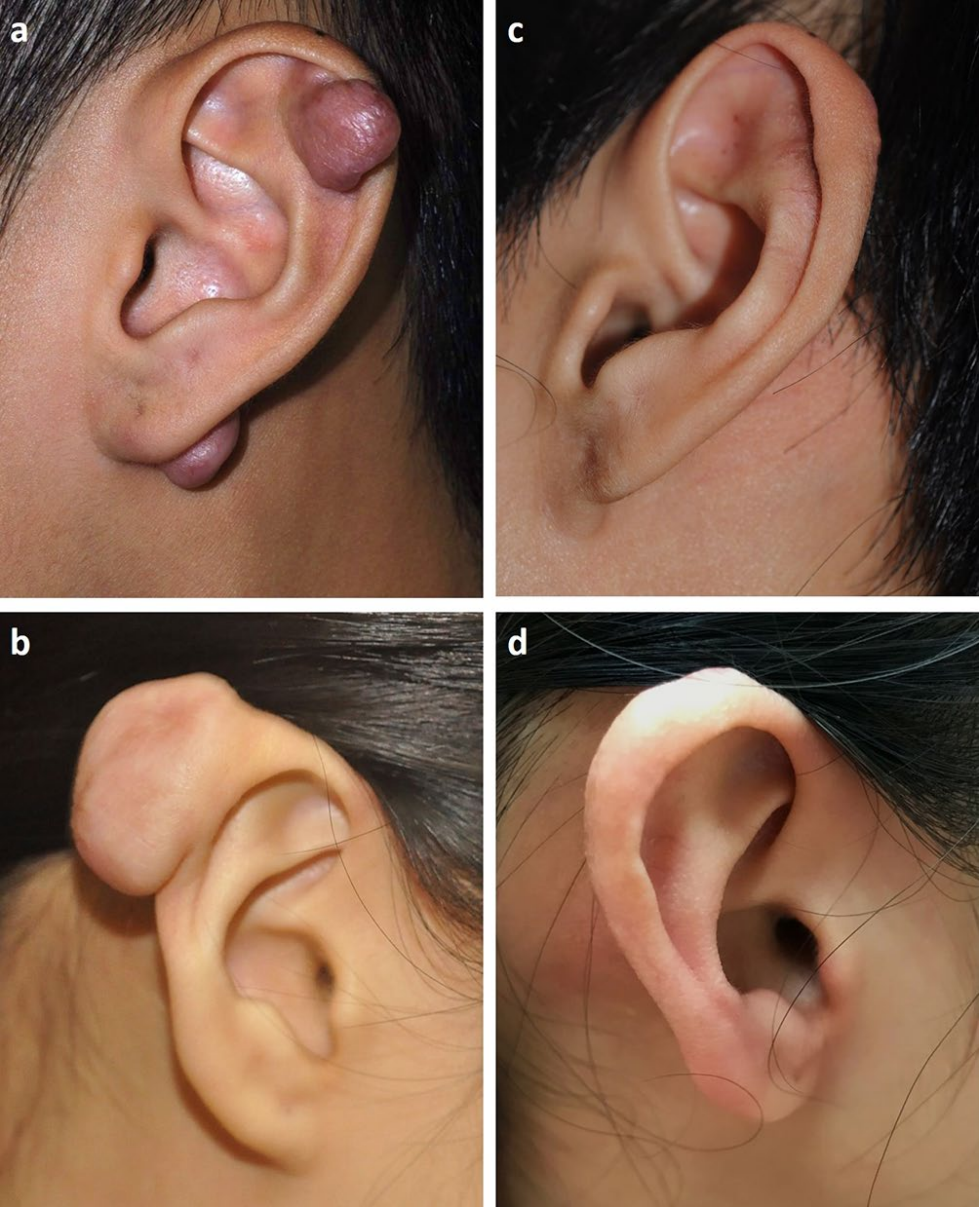
Clinical photographs of auricular keloids before and after treatment. (**a**) Two Chang-Park classification type I keloids located on the left ear helix and lobe. (**b**) Solitary large-sized Chang-Park classification type I keloid on the right ear helix. (**c**) The patient’s appearance in (**a**) after 20 months of treatment. (**d**) The patient’s appearance in (**b**) after 14 months of treatment.

The majority of patients were of Asian ethnicity, with only one Caucasian. The mean age was 27.1 years, ranging from 18 to 55 years. The location of keloids was divided into helix (including the cartilage part) and earlobe, with 32 keloids (71.1%) and 13 keloids (28.9%) for each, respectively (Table 1). The average dimension of these keloids (longitudinal length × horizontal width) was 1.8 cm × 1.2 cm, and the preoperative clinical photos were shown (Fig. 1a,b). There were 38 (84.4%), eight (17.8%), and four (8.9%) keloids treated previously with intralesional corticosteroid injection, surgery, and cryotherapy, respectively. According to Chang-Park classification, nine keloids were classified as type I, 30 for type II, two for type III, four for IV, and none for type V (Table 1).

**Table 1:** Characteristics of patients and auricular keloids.

	Mean (N)	SD (%)
Age (years)	27.1	7.5
**Sex (patients)**		
M	5	14.3
F	30	85.7
**Previous treatment (ears)**		
Surgery	8	17.8
Intralesional injection	38	84.4
Cryotherapy	4	8.9
None	7	15.6
**Size (cm)**		
Length × width	1.8 × 1.2	0.9 × 0.6
**Location of ear keloid (ears)**		
Ear helix	32	71.1
Ear lobe	13	28.9
**Chang-Park classification (ears)**		
I	9	20
II	30	66.7
III	2	4.4
IV	4	8.9

*F* female, *M* male, *N* number, *SD* standard deviation.

### Therapeutic outcome

The preliminary result showed keloid recurrences occurred in 3 of 45 (6.7%) (Table 2). One recurrence case was associated with pregnancy. The mean wound healing time was 2.4 weeks. There were no flap necrosis, surgical wound infection, or complications of corticosteroid injection and radiotherapy; only one case presented with seroma formation after 2.5 months, which subsided following incision and drainage. Clinical photographs of the postoperative auricular keloids taken during follow-up visits were shown (Fig. 1c,d).

**Table 2:** Treatment outcomes of auricular keloids.

	Mean (N)	SD (%)
**Radiation therapy**		
Dosage (cGY)	1578.6	133.5
Interval (ears)		
0,1,2 (day after OP)	20	44.4
0,1,4 (day after OP)	25	55.6
Wound healing (weeks)	2.4	0.7
Recurrence (ears)	3	6.7
Follow-up period (months)	24.1	12.6

*N* number, *OP* operation, *SD* standard deviation.

### Risk factors associated with recurrence

There were no significant differences in age, sex, previous treatment history, size, location at the ear, and type of Chang-Park classification among the auricular keloids with recurrences and without recurrences (Table 3). Radiation dosage was significantly higher (1584.6 cGy) in the auricular keloids without recurrences than that with recurrences (1500.0 cGy) (*p* = 0.001) (Table 3). Wound-healing time was similar in the keloids with recurrences and without recurrences.

**Table 3:** Risk factors of keloid recurrences.

	No recurrence (N = 42)		Recurrence (N = 3)		*p*-value
	Mean (N)	SD (%)	Mean (N)	SD (%)	
Age (years)	26.8	7.3	30.3	6.1	0.438
**Sex (patients)**					
M	5	11.9	0	0	0.447
F	37	88.1	3	100	
**Previous treatment (ears)**					
Surgery	8	19.0	0	0	0.999
Intralesional injection	35	83.3	3	100	
Cryotherapy	2	4.8	2	66.7	
None	7	16.7	0	0	
**Size (cm)**					
Length × width	1.9 × 1.2	0.9 × 0.6	1.5 × 0.8	0.9 × 0.3	0.472
**Location of ear keloid (ears)**					
Ear helix	30	71.4	2	66.7	0.999
Ear lobe	12	28.6	1	33.3	
**Chang-Park classification (ears)**					
I	9	21.4	0	0	0.900
II	27	64.3	3	100	
III	2	4.8	0	0	
IV	4	9.5	0	0	
**Radiation therapy**					
Dosage (cGY)	1584.6	136.8	1500	0	0.001
Interval (ears)					
0,1,2 (day after OP)	19	45.2	1	33.3	0.999
0,1,4 (day after OP)	23	54.8	2	66.7	

*F* female, *M* male, *N* number, *OP* operation, *SD* standard deviation.

## Discussion

This novel multimodal approach in a single session composed of keloid enucleation, reconstruction with a core fillet flap, intraoperative intralesional corticosteroid injection, and immediate postoperative radiotherapy is an optimal regimen to treat auricular keloids with a low recurrence rate and few complications associated with radiotherapy and long-term corticosteroid injection.

Auricular keloids are challenging for surgical resection due to the preservation of the three-dimensional contour of the external ear and the scarcity of tissue laxity^1^. The recurrence rate of core enucleation with a fillet flap was lower than that of total excision, owing to the avoidance of high tension and distortion of anatomic structures. The nourished blood supply of fillet flaps ensured no event of flap necrosis in our study^1^. Nevertheless, adjuvant therapies were often needed due to a high recurrence rate of surgical resection alone, ranging between 45% and 100%^7^. Intralesional corticosteroid injection and radiotherapy have been the most widely used regimens^8^. While the recurrence rate of radiotherapy was lower than that of intralesional corticosteroid injection^9,10^, irradiation could only damage the majority of keloid cells. Intralesional corticosteroid injections were needed to inhibit re-propagation of the residual irradiation-resistant keloid cells^11^.

Corticosteroids can inhibit the migration of activated fibroblasts and induce vasoconstriction to reduce inflammatory responses^12^. Intraoperative injections arrest the action of fibroblast within post-traumatic 24 hours, and injections after administration of local anesthetics could eliminate injection pain and physical challenge during injection due to the lesion density^11,13^. In Table 4^6,9,14^, the recurrence rates of the auricular keloids receiving intraoperative and postoperative intralesional corticosteroid injection ranged from 9.5% to 33.3%. Despite long-lasting results, the complications comprised wound dehiscence, depigmentation, flap necrosis, and abscess formation at the intralesional site^6,9,14^. The keloid tissues treated with corticosteroid previously were adhesive to make complete surgical resection difficult, and thus there was no advantage to corticosteroid injection before scheduled excision^15^.

**Table 4:** Comparison with previous literatures adopting surgical excision with intraoperative [Triamcinolone acetonide (40 mg/cc)] and postoperative intralesional corticosteroid injection.

	Patient (N)	Keloid (N)	Ethnicity	Age (years)	Sex	Size^a^ (cm)	Location	Prior OP	Prior IL	Surgery	Scheduled post-OP IL	Recurrence rate (keloid N)	Follow-up	Complication (keloid N)
1^14^	64	92	African American (83%) Caucasian (9%) Hispanic (8%)	28.3	F (78%) M (22%)	1.94	NA	25% (23/92)	NA	Total excision ± flap	Post-OP 1st and 2nd month	23.3% (10/43)	10.4 years in 43 keloids	5.4% (5/92); depigmentation (1), scar widening (1), and wound dehiscence (1)
2^9^	12	12	Black (67%) Caucasian (8%) Hispanic (25%)	29.4	F (33%) M (67%)	2.00	Lobe (100%)	50% (6/12)	25% (3/12)	Total excision	Post-OP 1st, 3rd, and 5th week	33.3% (4/12)	19 months	No
3^6^	15	21	NA	24.8	F (100%)	NA	Lobe (71.4%) Helix (28.6%)	13.3% (2/15)	20% (3/15)	Enucleation + core fillet flap	Post-OP 2nd week and monthly dependent on lesion	9.5% (2/21)	21.9 months	Flap necrosis and infection, abscess after IL, and neuroma after 2^nd^ OP
Our	35	45	Chinese (97.1%) Caucasian (2.9%)	27.1	F (85.7%) M (14.3%)	1.85	Lobe (71.1%) Helix (28.9%)	17.8% (8/45)	84.4% (38/45)	Enucleation + core fillet flap	No	6.7% (3/45)	24.1 months	2.2% (1/45); seroma (1)

^a^Size was shown with averaged longitudinal length.

*F* female, *IL* intralesional corticosteroid injection, *M* male, *N* number, *NA* not available, *OP* operation.

Irradiation could restore a balance between collagen degradation and synthesis by affecting extracellular matrix gene expression^8^. The recurrence rates of postoperative radiotherapy (Table 5)^16–20^ varied from 4% to 19%. Acute radiation-associated adverse events, including skin ulceration, delayed wound healing, and skin graft necrosis, as well as chronic complications including permanent color change and telangiectasia, were reported in these studies^16–21^. Radiation-based treatments for keloids consist of electrons, X-rays, and brachytherapy^22^. Although brachytherapy has the lowest recurrence rate, the radioactive source of brachytherapy (iridium-192) has not been widely available^23^. Electron beam irradiation was otherwise superior to X-ray in treating keloids^24^. In addition to radiation modality, radiation dosage, fractionation, and overall treatment time also determine the therapeutic effect^25–29^. There is a dose–response relationship in treating postoperative keloids, and the total dosage of 20 Gy to 30 Gy was suggested to achieve the lowest recurrence rate^25–28^. In our analyses, higher radiation dosage was associated with a lower recurrence rate (*p* = 0.001).

**Table 5:** Comparison with previous literatures adopting surgical excision with postoperative radiation therapy.

	Patient (N)	Keloid (N)	Ethnicity	Age (years)	Sex	Location	Prior OP	Surgery	External beam RT	RT regimen (total dose/fraction × dose per fraction) (Gy)	Recurrence rate (keloid N)	Follow-up (months)	Complication (keloid N)
1^16^	23	30	NA	28	F (75%) M (35%)	Lobe (100%)	33.3% (10/30)	Total excision	6-MeV electron	15/3 × 5; 1 Fr. in post-OP 2 h and 2 Fr. in post-OP 2–3 days	13.3% (4/30)	26	Hyperpigmentation 13.3% (4/30)
2^17^	57	63	NA	NA	F (91.2%) M (8.8%)	NA	0%	Total excision: 59% (37/63) Enucleation: 41% (26/63)	4-MeV electron	15/3 × 5; over post-OP 3 days	4.8% (3/63) (8.1% for total excision and 0% for enucleation)	18	No
3^18^	35	NA	NA	24	F (97%) M (3%)	Lobe (100%)	All with OP or IL	Total excision	100 kV X-ray photon	10/1 × 10; within post-OP 24 h	11.8% (4/34)^a^	60	Transient erythema and post-radiation hyperpigmentation
4^19^	145	174	Japanese	NA	NA	Lobe (100%)	13% (19/145)	Wedge excision + PS Simple excision + PS Simple excision + V–Y flap	4-MeV electron	High dose: 15/3 × 5; over post-OP 3 days (20%) Low dose: 10/2 × 5; over post-OP 2 days (80%)	4.0% (7/174) (4.7% for primary keloid and 0% for recurred keloid)	18	NA
5^20^	21	NA	African	22	F (77.5%) M (22.5%)	Lobe (72%) Helix (28%)	33.3% (7/21)	Total excision ± rhomboid flap	100 kV X-ray photon	12/3 × 4; each Fr. on post-OP 3rd, 4th, and 5th day	19.1% (4/21)^a^ (14.3% for primary keloid and 28.6% for recurred keloid)	18	Depigmentation and atrophy after IL (100%), infection (1), and flap necrosis (1)
Our	35	45	Chinese (97.1%) Caucasian (2.9%)	27.1	F (85.7%) M (14.3%)	Lobe (71.1%) Helix (28.9%)	17.8% (8/45)	Enucleation + core fillet flap	6-MeV electron	15/3 × 5 or 18/3 × 6; 1 Fr. in post-OP 24 h and 2 Fr. in post-OP 2–4 days	6.7% (3/45)	24.1	2.2% (1/45); seroma (1)

^a^Number of patients.

*F* female, *Fr.* Fraction, *h* hour, *IL* intralesional corticosteroid injection, *M* male, *N* number, *NA* not available, *OP* operation, *PS* primary suture, *RT* radiation therapy.

Nevertheless, there was a correlation between adverse effects and the total irradiation dose^25–28^. An optimal dose to reach a balance between local control and adverse effects for resected auricular keloids has not been well-established. We proposed a radiation regimen of 15 Gy or 18 Gy in three fractions completed within two or four days, enabling the transition of keloid fibroblasts from radioresistant status into the radiosensitive status and normal skin cells to repair^29^. Skin cancers arising from the post-radiation auricular keloids have not been reported in our and other studies.

The multimodal approach in our study possesses the advantages of intralesional corticosteroid injection and radiotherapy to achieve a better recurrence control without additional complications. There was no delayed wound healing in a single session of intralesional injection. Routine postoperative injections were abandoned to reduce the side effects of long-term intralesional corticosteroid injections, including skin and subcutaneous fat atrophy, telangiectasia, pigmentary change, skin necrosis, and ulceration. In our study, there were no radiation-related complications, especially radiation-induced hyperpigmentation, which may be partly attributed to additional intraoperative corticosteroid injection, effective in treating radiation dermatitis^30^. Another similar study utilizing individualized surgery (simple excision, core excision with scar flap reconstruction, and complete keloid excision with adjacent flap plasty) following immediate corticosteroid injection and radiotherapy to treat auricular keloids showed a recurrence rate of 12.39%^31^. Although a high total irradiation dose (20 Gy) and additional three postoperative monthly injections were applied, the recurrence rate was not superior to our results.

## Limitations

This is a retrospective single center study, and further large-scale randomized controlled trial with an extended follow-up period is needed to confirm the superiority of this method compared to other conventional treatments. Despite these limitations, this novel multimodal approach can serve as a therapeutic option for auricular keloids.

## Conclusion

In conclusion, we demonstrated that enucleation and core fillet flap followed by intraoperative intralesional corticosteroid injection and immediate postoperative radiotherapy obtained a low recurrence rate and minimal complications. This novel multimodal regimen in a single session is an effective and safe treatment for auricular keloids.
